# Erythroderma and Skin Desquamation in Paederus Dermatitis

**DOI:** 10.1155/2021/7257288

**Published:** 2021-12-28

**Authors:** Thitinan Kitisin, Passanesh Sukphopetch

**Affiliations:** Department of Microbiology and Immunology, Faculty of Tropical Medicine, Mahidol University, Bangkok 10400, Thailand

## Abstract

Exfoliative erythroderma is rare but serious condition, which requires close supervision. We report a rare case of 28-year-old man with kissing lesions of Paederus dermatitis at his right side of neck. The rash caused by *Paederus* beetle was improved after treatments. However, the patient developed generalized erythema with desquamation and scaling. The patient was successfully treated topically with moisturizing liquid soap and topical moisturizer with emollients and humectants, and triamcinolone lotion was applied on the bright red lesion. The patient was also treated with oral replacement solution and tropical azelaic cream was applied on the hyperpigmented kissing lesion. This case report shows the importance of a diagnostic practice with follow-up examination.

## 1. Introduction

Paederus dermatitis is one type of irritant contact dermatitis that occurs worldwide, especially in tropical and subtropical regions. Most of the patients develop localized blistering and inflammation after contacting with *Paederus* beetles (family *Staphylinidae*). The lesion is local and unlikely to develop generalized abnormal skin presentations [1]. We report an unusual exfoliative erythroderma of Paederus dermatitis.

## 2. Case Presentation

**Figure 1:**
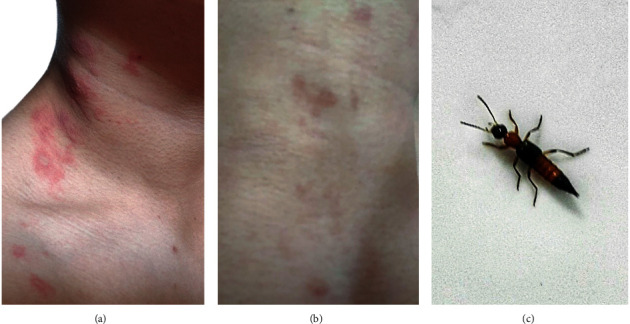
A typical burnt-like lesion of Paederus dermatitis. (a) Linear well-defined erythematous and edematous plaques were presented at patient's right side of neck. (b) Complete resolution with small degree of hyperpigmentation was observed within 14 days. (c) A photo of suspected *Paederus* beetle was taken by the patient.

**Figure 2:**
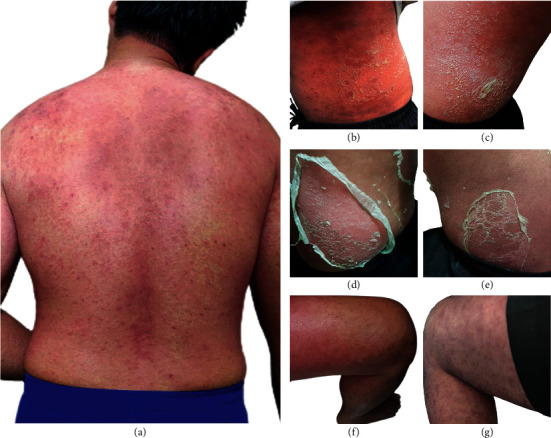
Generalized erythroderma with desquamation and scaling was shown around the (a) back, (b–e) body, and (f, g) legs of the patient at day 5 of follow-up.

A 28-year-old man visited to the dermatological unit with linear well-defined erythematous and edematous plaques associated with burning sensation and slight itching at his right side of neck (Figure 1(a)). Examination of the lesions revealed the typical of kissing lesions of Paederus dermatitis for more than 24 hours after contact with the *Paederus* beetle (Figure 1(c)). The patient was treated with topical fusidic acid with betamethasone, oral nonsedating antihistamine, fexofenadine 180 mg once a day, and wet dressings. Notably, this patient has had a history of fexofenadine and topical ointment uses without any signs and symptoms of drug allergy. At 5-day follow-up, the rash caused by *Paederus* beetle was improved, the erythematobullous lesions were decreased but facial, body, arms, legs, and sole areas had developed generalized erythema with desquamation and scaling (Figures 2(a)–2(g)).

**Figure 3:**
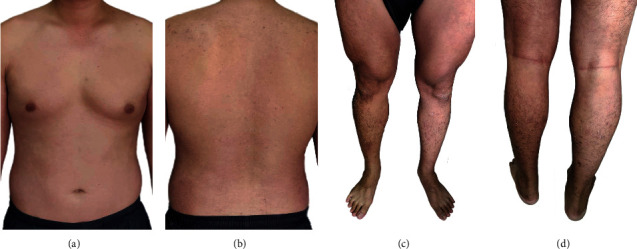
Recovery of the skin lesions from generalized erythema at the upper body: (a) front and (b) back and lower body: (c) front and (d) back were close to normal.

In-depth careful history taking and physical examination were done to determine the factors associated with exfoliative erythroderma, such as drug-induced desquamating skin rash and past history of erythrodermic and pustular psoriasis, including family history of cancer. The results revealed no history associated with those relative risks. Tumor marker screenings including CEA, PSA, and AFP in this patient were negative. Results from complete blood count, chest radiograph, and liver function test appeared normal. No typical pathogenic bacteria or fungi was detected. Therefore, the physician diagnosed an exfoliative dermatitis after exposure to *Paederus* beetle without other medical or cancer risks. The patient was treated for 7 days with oral rehydration solution an ceramide-containing skin care with emollients and humectants, and triamcinolone lotion was applied on the bright red lesion. The patient was also treated with tropical azelaic cream for reducing hyperpigmentation at kissing lesions. The patient was consulted online with the physician every day and followed up at the clinic once a week. The consultation was continued for 2 weeks. The treatments led to complete resolution within 14 days with small degree of hyperpigmentation since his first visit (Figure 1(b)). Clinical follow-up 15 weeks later was done to exclude erythrodermic and pustular psoriasis in this patient and no remission was observed. Recovery of the skin lesions from generalized erythema was closed to normal (Figures 3(a)–3(d)). Clinical follow-up was continued once a month for 6 months. No signs of erythrodermic psoriasis as well as recurrent erythroderma were observed. Moreover, symptoms of erythroderma in this patient with no rash on the palms/soles of the feet were reminiscent of less erythrodermic psoriasis.

## 3. Discussion

Paederus dermatitis is an acute irritant contact dermatitis caused by accidently crushing the *Paederus* beetles which release a haemolymph fluid called paederin (C_24_H_43_O_9_N) [1]. Although 50 of more than 600 described species of *Paederus* have been associated with Paederus dermatitis worldwide, however, *Paederus fuscipes* (*Coleoptera*: *Staphylinidae*) was mostly reported as the causative agent in Thailand [2]. These nocturnal *Paederus* beetles have often attracted themselves to incandescent and fluorescent lights, which may explain why the patients especially from the urban area who sleep with light on have Paederus dermatitis [3].

On crushing the insect, patients are being exposed to paederin and experience self-limiting acute severe skin irritation, erythema, vesiculation, and crusting. Periorbital dermatitis and keratoconjunctivitis were less commonly reported [4]. Moreover, generalized erythroderma with desquamation is an atypical variant of Paederus dermatitis, which has been previously reported predominantly on the upper body part [5]. A previous *in vivo* study has revealed that paederin enhances the expressions of toll-like receptor (TLR)-2 and interleukins (IL)-4, -5, and -13, which may be relevant to the disease severity in patients [6].

Treatment of Paederus dermatitis is initially done by washing the irritant area with soap and water, using cold wet compression and application of topical steroid. Preventing human-beetle contact is the primary prevention strategy by turning off the light and closing all windows and doors securely before sleep [7].

## 4. Conclusions

We present atypical case of Paederus dermatitis as generalized erythroderma with desquamation. Further study is required to elucidate the molecular mechanisms related to exfoliative dermatitis in Paederus dermatitis patients.

## Data Availability

The data used to support the findings of this study are available from the corresponding author upon request.

## Abbreviations

CEA: Carcinoembryonic antigen
PSA: Prostate-specific antigen
AFP: Alpha-fetoprotein
TLR: Toll-like receptor
IL: Interleukins.
